# Sequential Grouping Modulates the Effect of Non-Simultaneous Masking on Auditory Intensity Resolution

**DOI:** 10.1371/journal.pone.0048054

**Published:** 2012-10-24

**Authors:** Daniel Oberfeld, Patricia Stahn

**Affiliations:** Department of Psychology, Section Experimental Psychology, Johannes Gutenberg-Universität Mainz, Mainz, Germany; Baycrest Hospital, Canada

## Abstract

The presence of non-simultaneous maskers can result in strong impairment in auditory intensity resolution relative to a condition without maskers, and causes a complex pattern of effects that is difficult to explain on the basis of peripheral processing. We suggest that the failure of selective attention to the target tones is a useful framework for understanding these effects. Two experiments tested the hypothesis that the sequential grouping of the targets and the maskers into separate auditory objects facilitates selective attention and therefore reduces the masker-induced impairment in intensity resolution. In Experiment 1, a condition favoring the processing of the maskers and the targets as two separate auditory objects due to grouping by temporal proximity was contrasted with the usual forward masking setting where the masker and the target presented within each observation interval of the two-interval task can be expected to be grouped together. As expected, the former condition resulted in a significantly smaller masker-induced elevation of the intensity difference limens (DLs). In Experiment 2, embedding the targets in an isochronous sequence of maskers led to a significantly smaller DL-elevation than control conditions not favoring the perception of the maskers as a separate auditory stream. The observed effects of grouping are compatible with the assumption that a precise representation of target intensity is available at the decision stage, but that this information is used only in a suboptimal fashion due to limitations of selective attention. The data can be explained within a framework of object-based attention. The results impose constraints on physiological models of intensity discrimination. We discuss candidate structures for physiological correlates of the psychophysical data.

## Introduction

The intensity of an auditory stimulus is one of the most important basic attributes of auditory perception, besides pitch and spatial localization. We use information about auditory intensity in many different situations. If we want to cross a road, the acoustic intensity (change) provides information about the distance or the time-to-contact of an approaching car (e.g., [1]). We attend to prosodic highlighting [2] when trying to grasp the meaning of a spoken sentence or to learn a language (e.g., [3]). In the context of music perception and performance, accented tones play an important role for the perception of rhythm and musical meter (e.g., [4]).

**Figure 1:**
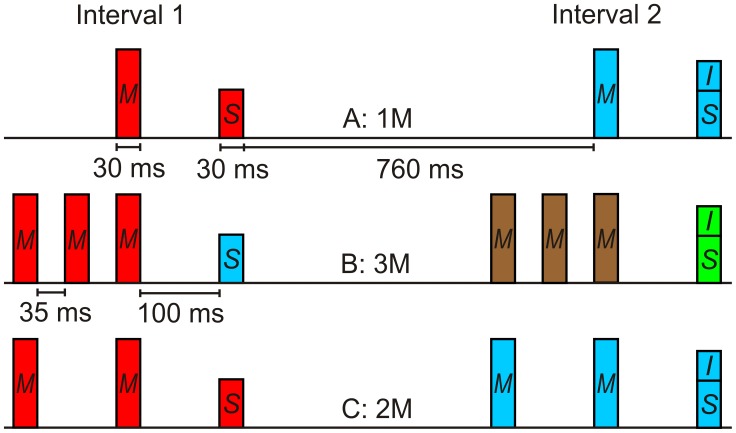
Schematic depiction of the stimulus configurations used in Experiment 1 (grouping by temporal proximity). Two-interval intensity discrimination task presenting the standard (*S*) in one interval and the standard-plus-increment (*S+I*) in the other interval. The position of the standard-plus-increment was randomly selected. Listeners decided in which interval the louder target tone had been presented. Panel A: one forward masker (*M*) per observation interval (1 M). Panel B: three maskers (3 M). Panel C: two maskers (2 M). The colors indicate the expected grouping. We expected the masker(s) and the target presented within an observation interval to be grouped together in the 1 M and 2 M conditions, but to be perceived as separate auditory objects in the 3 M condition. Not shown is the in-quiet condition presenting no masker. The displayed tone durations include 5 ms on- and off ramps.

For sounds presented in quiet, the intensity resolution of normally hearing subjects is rather high (e.g., [5]). To a large extent, the intensity resolution for sounds presented in isolation or together with a simultaneous masker can be explained by physiological mechanisms in the cochlea and the auditory nerve (e.g., [6]), as for example basilar membrane compression. Models based on these principles are capable of representing a significant portion of the psychoacoustic data (e.g., [7]). However, for sounds presented in a temporally more complex acoustic context, as it applies to many environmental sounds, the intensity resolution can be much lower than for sounds in quiet (e.g., [8]), with intensity difference limens (DLs) being elevated by up to 20 dB. This study deals with intensity discrimination under non-simultaneous masking, e.g., when the target tone is combined with a forward masker preceding it by 100 ms. Quite surprising, a rather complex pattern of effects on intensity resolution is observed in this situation (e.g., [8], [9], [10]), although it represents only a minor increase in temporal complexity compared to intensity discrimination in quiet. Identifying the mechanisms responsible for the sometimes dramatic impairment in performance caused by the maskers has proven to be difficult (cf. [10]). The aim of this study was to test the hypothesis that the strong impairment in intensity resolution caused by non-simultaneous maskers can, at least in part, be attributed to the failure to selectively attend to the target tones and to ignore the maskers. To this end, we compared intensity resolution under non-simultaneous masking in two types of conditions. In the first type of conditions, the sequence of maskers and targets was constructed in a way that favored the perceptual grouping of the maskers and the targets into two separate auditory objects. Phenomenologically, an auditory object can be defined as sound elements that are perceived as belonging together, and as separated from other sound elements [11], [12], [13], [14]. We expected the detrimental effect of the maskers to be smaller in these conditions than in the second type of conditions where maskers and targets were expected to be grouped together on the basis of temporal proximity (e.g., [15]). Thus, the present study explored the potential role of *object-based selective attention* in an intensity discrimination task. Research on object-based attention in the visual domain (e.g., [16], [17], [18], [19], [20], [21]) has demonstrated that it is more difficult to selectively attend to a feature *within* an object than to attend to one object and ignore another object. In the auditory domain, there is a long tradition of research on “auditory scene analysis” [22], which is concerned with the question of how the acoustic input reaching a listener’s ear is structured into different “objects”, “streams”, or “groups”. For example, using the famous example of a cocktail party [23], you want to selectively attend to the speech signals produced by the person you are listening to (i.e., the “target stream”), while trying to ignore the hubbub of other conversations around you. Returning to the less complex stimulus configuration used in an intensity discrimination task with non-simultaneous maskers, we tested the hypothesis that the *perceptual grouping* of the target and the masker(s) into separate objects (cf. [12], [13]) facilitates selective attention to the target and therefore reduces the masker-induced impairment in intensity discrimination. In the usual forward-masked two-interval intensity discrimination task, the masker-target pair presented in for example the first observation interval (see **Figure 1**, Condition A) can be expected to be grouped together on the basis of temporal proximity (e.g., [15], [24], [25]). Thus, masker and target are likely perceived as one unitary auditory object [11], [13]. Therefore, selectively attending only to the target intensity while ignoring the masker should be more difficult than if masker and target were perceptually organized as two *separate* auditory objects (cf. [13], [14]). In fact, many subjects in our previous experiments on forward-masked intensity discrimination [10], [26], [27], [28] reported to hear the target tone presented shortly after an intense masker as a weak “echo”, which would be a clear example of perceiving the two tones as one unitary object.

Concerning the terminology it should be noted that Alain and Arnott [14] suggested to distinguish between auditory sources, events, objects, and streams, although in the literature these concepts are often used in an interchangeable fashion [11], [29], [30], [31], [32], and several partly overlapping and partly differing concepts for auditory objects have been proposed [11], [12], [13], [14]. For the experimental manipulations we used, the potential distinctions between auditory objects, groups, and streams are not critical. We simply aimed at presenting conditions where the maskers were perceived as belonging together, and as segregated from the targets. In fact, auditory object formation in the sense that acoustical elements belonging to this “object” are processed as an entity such that for example comparisons are easier within the “object” than across different “objects” have been reported for sequential grouping by temporal proximity (e.g., [25], [33]), sequential streaming (e.g., [34]), and object formation on the basis of spectral or binaural cues (e.g., [35]). We use the terms *object* and *object-based attention* because they are very well established in the visual domain, and not to imply that we are referring to something conceptually different than auditory streaming or auditory grouping.

Before introducing the two experiments, we provide a brief description of effects of non-simultaneous masking on intensity resolution, and of models proposed for these effects. If a brief target sound is presented together with temporally non-overlapping maskers auditory intensity resolution can be strongly impaired (e.g., [8], [10], [36], [37]). As an example, consider intensity DLs for a brief (30 ms) pure-tone standard presented at an intermediate sound pressure level (about 60 dB SPL). In quiet, the DL is only about 1 dB (e.g., [5]). However, an intense on-frequency forward or backward masker (about 90 dB SPL) separated from the target by a silent interval of about 100 ms has been reported to cause DL-elevations of up to 20 dB [8]. An important result is that the same intense masker causes only small DL-elevations for standards presented at high levels (e.g., 90 dB SPL), but surprisingly also at low levels (e.g., 30 dB SPL). Thus, with an intense masker the DL-elevation is maximal at intermediate rather than at low standard levels. In addition to this *midlevel hump* [36], there is evidence for a *mid-difference hump* [10]. For a fixed standard level, the masker-induced DL-elevation was found to be strongest at intermediate level differences between masker and standard.

Several explanations have been proposed for these phenomena (for an in-depth discussion see [10]). Zeng and colleagues proposed an elegant model based on adaptation in the auditory nerve [36], referring to the slow recovery from prior stimulation in low spontaneous-rate (SR) auditory nerve neurons. Yet, subsequent experiments demonstrated that the effects of backward maskers on intensity resolution are at least as strong as for forward maskers (e.g., [8]), although at masker-target intervals longer than a few milliseconds backward maskers should not alter the neural representation of target intensity in the auditory nerve (for a detailed discussion see [28]). In a similar vein, DL elevations caused by contralaterally presented forward maskers [38] are incompatible with mechanisms at the earliest auditory processing stages. For this reason, explanations based on more central processes have been proposed [10], [37], [39]. According to the *referential encoding hypothesis* [37], [39], the masker presented between the targets in a two-interval task degrades the sensory memory trace of the target presented in the first observation interval (cf. [40]). The *loudness enhancement hypothesis* [37] attributes the masker-induced DL-elevations to variability in the loudness representation of the target, induced by systematic changes in target loudness caused by the masker (cf. [26]). The fact that several rather different models have been proposed for the effects of non-simultaneous masking on intensity resolution indicates that it is a challenging task to explain the psychophysical data. In fact, none of the three models introduced above is capable of accounting for the complete range of empirical findings (cf. [10], [27]). To give two examples, according to the assumptions underlying the referential encoding hypothesis [37], [39], a midlevel hump should be observed in a one-interval intensity discrimination task in quiet (cf. [10]), but this is not the case (e.g., [41]). The loudness enhancement hypothesis [10], [37], on the other hand, predicts that the listeners use a weighted average of masker and target loudness when making their decision (“energy detection”), incompatible with the observation of *negative* decision weights assigned to masker intensity at some masker-target level combinations [27].

It is important to note that all of the previous models assume that the representation of target intensity used in the decision process is degraded by the maskers, either in the auditory nerve, or at higher processing stages. The framework we propose here differs from previous explanations because it takes into account the possibility that a precise representation of target intensity is available at the decision stage, but that this information is used only in a suboptimal fashion, because task-irrelevant information about masker intensity is factored into the decision. According to our hypothesis, this is due to a failure to selectively attend to the targets while ignoring the maskers. In fact, intensity information from to-be-ignored forward maskers systematically influences the decision in an intensity discrimination task [27].

In the first experiment, each target in a two-interval intensity discrimination task was preceded by either one, two, or three forward maskers. The temporal sequence of masker(s) and targets was selected so that either (a) the masker(s) and the target presented in each interval could be expected to be grouped together on the basis of temporal proximity [42], or (b) that the maskers could be expected to be processed as one auditory object, and the target as a separate object [25]. In case (a), it should be difficult to selectively attend to the target and to ignore the masker. In case (b), selectively attending to the target tones should be facilitated. Note that previous research has demonstrated that grouping by temporal proximity can constitute auditory objects [24], [33], so that for example comparisons within a temporal group are easier than between temporal groups [33].

In Experiment 2, we presented a *streaming condition* where the two target tones were embedded in an isochronous sequence of eight masker tones. We expected the maskers to be perceived as one auditory stream, and the two target tones as separate auditory events. The remaining conditions were control conditions in which no regular masker rhythm was presented. Thus, we assumed the target and its adjacent masker(s) to be grouped together. Our hypothesis was that the masker-induced DL elevation would be smaller in the streaming condition than in the remaining conditions. The aim of the second experiment was simply to rule out the possibility that the effect of grouping the maskers and the targets as separate objects is idiosyncratic to the condition with three forward maskers studied in Experiment 1.

Both experiments included the standard level as an additional variable to investigate the mid-level hump, i.e. the more pronounced effect of non-simultaneous masking for a mid-level standard compared to a low-level standard. We expected a stronger release from masking in conditions facilitating selective attention to the targets for midlevel standards compared to low-level standards.

## Materials and Methods

### Ethics Statement

The experiments were approved by the ethics committee of the Department of Psychology, Universität Mainz. All subjects participated voluntarily after providing informed written consent. They received partial course credit or were paid €6 per hour. The experiments were conducted according to the principles expressed in the Declaration of Helsinki.

### Participants

Eight subjects (5 female, 3 male; aged 20–28 years) participated in Experiment 1. Seven subjects (4 female, 3 male; aged 20–28 years) participated in Experiment 2, one of them had already been tested in Experiment 1. All subjects were screened for normal hearing (within 10 dB) on the ear tested for frequencies between 250 and 4000 Hz. They were also screened for detection thresholds lower than 23 dB SPL in any masking condition, to ensure that the standard in the intensity discrimination task was always at least 7 dB SPL above threshold.

### Stimuli and Apparatus

In Experiment 1, the standard and the masker were 1 kHz pure tones with a steady-state duration of 20 ms, gated on and off with 5 ms cos^2^-ramps. In Experiment 2, the standard was a 1 kHz pure tone with a steady-state duration of 20 ms. The maskers were presented with a longer steady-state duration (50 ms) than the targets and at a different frequency (935 Hz, half an equivalent rectangular bandwidth below target frequency; [43]) in order to promote streaming [44], and so that the listeners could be instructed simply to compare the short tones (targets) and to ignore the longer tones (maskers). All tones were gated on and off with 5 ms cos^2^-ramps. The targets (standard and standard-plus-increment) were marked by visual signals.

The stimuli were generated digitally, played back via one channel of an RME ADI/S D/A converter (*f*_s_ = 44.1 kHz, 24-bit resolution), attenuated by a TDT PA5 attenuator, buffered by a TDT HB7 headphone amplifier, and presented to the right ear via Sennheiser HDA 200 circumaural headphones calibrated according to IEC 318 [45]. The experiment was conducted in a double-walled sound-insulated chamber.

### Conditions

In Experiment 1, two standard levels (30 and 60 dB SPL) were presented in quiet, and in three conditions presenting 90 dB SPL forward maskers. We included two different standard levels in order to investigate how perceptual grouping influences the midlevel-hump pattern, that is, a stronger masker-induced DL-elevation at the 60 dB SPL than at the 30 dB SPL standard level [36]. Although the definition of the midlevel-hump implies both a lower and upper comparison we did not include a high-level standard to save experimentation time because without exception all previous studies showed very small DL-elevations at high standard levels. We expected a stronger beneficial effect of perceiving the maskers and the targets as separate objects at the 60 dB SPL standard level, where with the usual stimulus configuration the masking effects are more pronounced.

In the condition with one masker per observation interval (1 M, see **Figure 1**A), the silent interval between masker offset and standard onset was 100 ms, corresponding to an IOI of 130 ms. In the 1 M condition, the two sounds presented in each interval (masker and target) were expected to be grouped together on the basis of temporal proximity [42]. Thus, we expected the masker and the target to be processed as a unitary auditory object, making it difficult to selectively attend to the target and to ignore the masker. In the 3 M condition (**Figure 1**B), three identical maskers were presented per interval, with IOIs of 65 ms separating them. The IOI between the third masker and the target tone was again 130 ms. Therefore, the three maskers were expected to be grouped together on the basis of temporal proximity. As a consequence, within each observation interval the three maskers were assumed to be processed as one auditory object, and the target as a separate object [25]. Compared to the 1 M condition, selectively attending to the target tones should thus be easier, and the masker-induced DL elevation should be smaller than in the 1 M condition. The condition with two maskers (2 M; **Figure 1**C) was identical to the 3 M condition, except that the middle masker was omitted. In the 2 M condition, the inter-onset interval (IOI) between the two maskers and the IOI between the second masker and the target were identical. Therefore, the temporal configuration did not encourage grouping the two maskers together and perceiving the target as a separate event, and we expected the DLs in this condition to be similar to the DLs in the 1 M condition.

Note that the frequency and the duration of the target and the maskers remained constant across conditions, as well as the temporal separation between the target and its adjacent masker. Therefore, according to current models for forward masking, in the auditory periphery at the most a weak summation of masking should occur in the 3 M condition [7], [46]. Therefore, the expected *reduction* in masking in the intensity discrimination task in the 3 M condition cannot be attributed to a reduction in peripheral adaptation.

**Figure 2:**
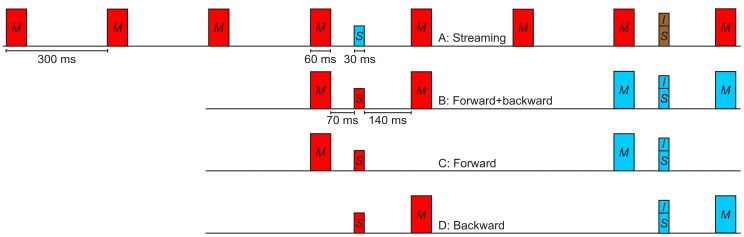
Schematic depiction of the stimulus configurations used in Experiment 2 (streaming). In the *streaming condition* (Panel A), the two target tones (standard and standard-plus-increment) were embedded in an isochronous sequence of eight maskers. We expected the maskers to be perceived as one auditory stream, and the two targets as separate auditory objects, as indicated by the colors. In the remaining conditions (Panels B to D), we expected each target to be grouped together with its adjacent masker(s). Not shown is the in-quiet condition containing no maskers. The tone durations include on- and off ramps.

In Experiment 2, two standard levels (30 and 60 dB SPL) were presented in quiet, and in four conditions including 85 dB SPL maskers. In the *streaming condition* (**Figure 2**A), the two target tones were embedded in an isochronous sequence of maskers (IOI = 300 ms). Four maskers were presented before the first target in order to allow for build-up of streaming (e.g., [47], [48]). We expected stream segregation on the basis of the loudness-, duration-, and pitch-differences between maskers and targets [49]. Note that mismatch negativity (MMN) research would describe the target as a triple-deviant relative to the maskers (e.g., [50]).

In the conditions presenting only a *forward masker* (**Figure 2**C) or only a *backward masker* (**Figure 2**D) we expected each target and its adjacent masker to be grouped together, causing difficulty in selectively attending to the target. Compared to the forward- or backward-masking conditions, the streaming condition might result in an increase or decrease in the DL elevations simply because each target tone was presented with two rather than only one adjacent masker. Therefore, we included the *forward-backward* masking condition (**Figure 2**B) which made it possible to determine the combined effect of two adjacent maskers, in the absence of a regular “masker rhythm” expected to result in streaming.

In the *in quiet* condition, no masker was presented. Note that all control conditions in Experiment 2 were generated simply by deleting maskers from the streaming condition. Therefore, the timing of the individual elements (e.g., the silent interval between the first and the second target) remained constant across all conditions. In both experiments, a within-subjects design was used. Each subject was tested in all experimental conditions.

### Procedures

Intensity DLs were measured in a two-interval, two alternative forced-choice task using an adaptive procedure with a 3-down, 1-up rule [51]. An intensity increment was added in-phase to the standard in one of the intervals (selected randomly). Listeners selected the interval containing the louder target tone (that is, the standard-plus-increment) and were instructed to ignore the maskers. Visual trial-by-trial feedback was provided.

The initial level of the intensity increment was 10 log_10_(Δ*I*/*I*) = 8 dB. The step size was 5 dB until the fourth reversal, and 2 dB for the remaining reversals. A track ended when 12 reversals had been obtained or when 70 trials had been presented. It was discarded if the standard deviation of 10 log_10_(Δ*I*/*I*) at the counting reversals was greater than 6 dB.

In Experiment 1, only one adaptive track was presented per experimental block. The arithmetic mean of 10 log_10_(Δ*I*/*I*) at the fifth reversal up to the last even-numbered reversal was taken as the DL estimate. Per listener and condition, six blocks were obtained in separate sessions. The order of conditions was randomized. For a given listener and condition, DL estimates more than 1.5 times the interquartile range beyond the quartiles were classified as outliers [52], resulting in the exclusion of at most two blocks per listener and condition. The same rule was applied for Experiment 2, and for the detection task.

In Experiment 2, each block contained two adaptive tracks presenting the same condition, which were run in a randomly interleaved fashion in order to reduce the predictability of the sequence of target levels [51]. A track ended when 12 reversals had been obtained or when 80 trials had been presented. If one of the tracks had already ended (because 12 reversals had occurred) before the termination of the other track, it was still presented with an a priori probability of 0.25. For each of the two tracks, the arithmetic mean of 10 log_10_(Δ*I*/*I*) at the fifth reversal up to the last even-numbered reversal was computed. The arithmetic mean of the two resulting values was taken as the DL estimate. A block was discarded if the standard deviation of 10 log_10_(Δ*I*/*I*) at the counting reversals was greater than 6 dB in either track. At least six blocks containing two tracks were obtained per listener and condition, in separate sessions.

Detection thresholds for the standard presented in the discrimination task (1 kHz, 30 ms including 5 ms cos^2^-ramps) were measured for the same experimental conditions as in the discrimination task. In one of the two observation intervals (selected randomly) the signal was presented. The other interval contained no signal. The observation intervals were marked by visual signals. The level of the signal was adjusted by a 3-down, 1-up adaptive rule. Listeners selected the interval containing the signal and were instructed to ignore the maskers. Visual trial-by-trial feedback was provided. The initial signal level was 30 dB SPL. The step size was 8 dB until the fourth reversal, and 2 dB for the remaining eight reversals. The arithmetic mean of the signal levels at the final eight reversals was taken as the detection threshold. A block was discarded if the standard deviation of the signal levels at the eight final reversals was greater than 6 dB. In Experiment 1, at least three blocks were presented per listener and condition. In Experiment 2, at least six blocks were obtained in separate sessions for each listener and each condition containing maskers, and at least four blocks in quiet.

At the end of the final session of Experiment 2, the perceived organization of the stimuli presented in the intensity discrimination task in this experiment was assessed via a questionnaire. On the questionnaire, different potential groupings of the tones presented within a trial were shown, using a graphical depiction of the temporal sequence of tones similar to **Figure 2**. In the figures, rectangles enclosing individual tones or groups of tones indicated different perceived configurations, with maskers and targets either grouped together, or grouped as separate units. For example, for the forward-masking condition (**Figure 2**, B), the drawings showed 1) the masker and target presented in each interval grouped together (grouping by temporal proximity), or 2) each single tone as one separate unit (no sequential grouping). Listeners received example trials from each condition (masking condition × standard level). After listening to the example trials for a given condition, listeners selected the alternative most accurately representing the perceived organization. They also had the opportunity to draw in the perceived organization on a schematic depiction showing only the tones but no rectangles indicating grouping, for the case that none of the variants we proposed provided a good description of their perception.

## Results

### Experiment 1: Maskers Grouped Together by Temporal Proximity

**Figure 3:**
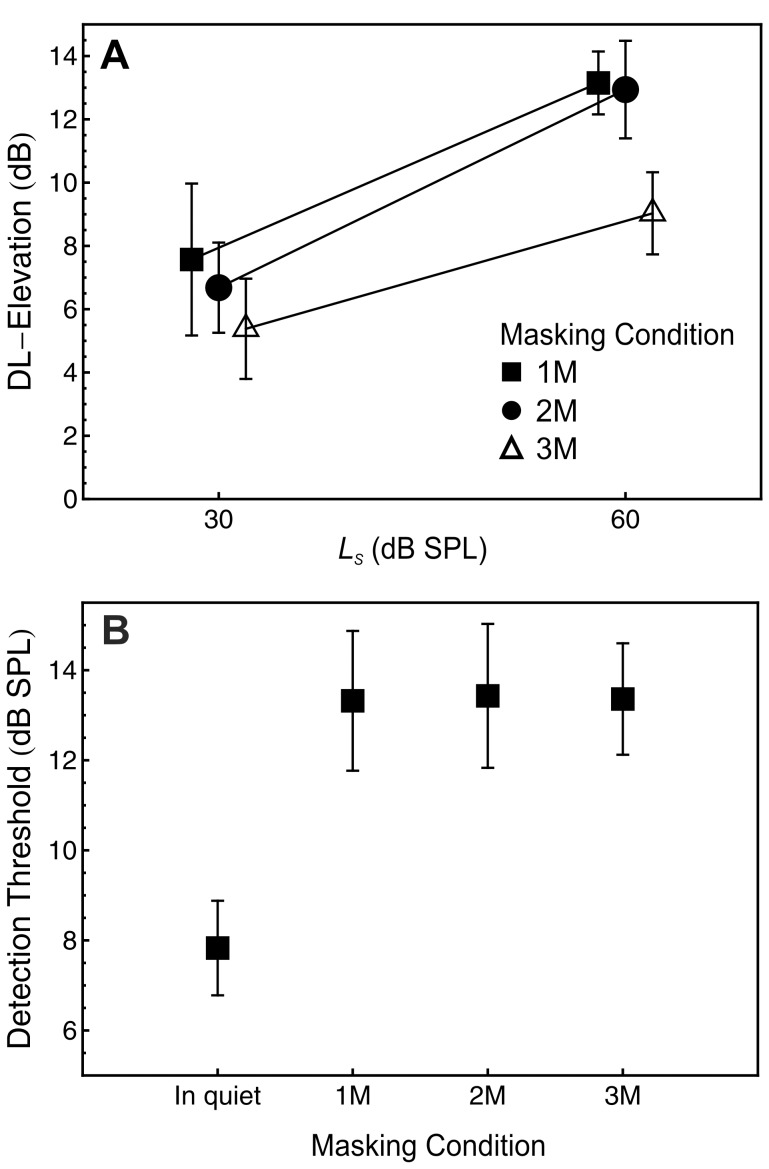
Experiment 1: intensity resolution and detection thresholds. Panel A: mean DL-elevation defined as the DL under masking minus the DL in quiet, as a function of standard level (*L*_S_) and number of maskers. Boxes: one masker (1 M). Circles: two maskers (2 M). Open triangles: three maskers (3 M). Panel B: Mean detection thresholds in quiet and in the three masking conditions. Error bars show plus and minus one standard error of the mean (SEM). *N* = 8.

The data were analyzed in terms of the DL-elevation, which denotes the difference between the DL, measured in units of 10 log_10_(Δ*I*/*I*), under masking and the DL in quiet. As **Figure 3**A shows, the DL-elevation was maximal for the one-masker (1 M) condition at both standard levels, followed by the two-maskers (2 M) condition. The smallest DL-elevation was observed in the three-maskers (3 M) condition, at both standard levels. Additionally, the DL-elevation was smaller for the 30 dB SPL standard than for the 60 dB SPL standard in all masking conditions, showing a midlevel-hump pattern.

The effects of standard level and masking condition on the DL-elevation were analyzed via repeated-measures analyses of variance (ANOVAs) using a univariate approach with Huynh-Feldt correction for the degrees of freedom. Partial η^2^ is reported as a measure of association strength. Post-hoc analyses of the differences between the masking conditions were computed by means of separate paired-samples *t*-tests (non-pooled error terms; [53]) and using Hochberg’s [54] sequentially acceptive step-up Bonferroni procedure which controls the Type I error rate.

The effect of masking condition was significant, *F*(2, 14) = 5.11, *p* = .022, 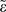 = 1.00, η^2^ = .422, confirming the descriptive differences between the three masking conditions. The DL-elevation was 3.15 dB (*SD* = 2.56 dB, Cohen’s [55] *d_z_* = 1.23) smaller in the 3 M condition than in the 1 M condition, 2.61 dB (*SD* = 3.08 dB, *d_z_* = 0.85) smaller in the 3 M condition than in the 2 M condition, and 0.55 dB (*SD* = 3.26 dB, *d_z_* = 0.17) smaller in the 2 M condition than in the 1 M condition. Post-hoc pairwise comparisons showed that the difference in the DL-elevation was significant between the 3 M and the 1 M condition, but not for the remaining pairs.

The effect of standard level was significant, *F*(1, 7) = 8.51, *p* = .022, η^2^ = .549, confirming the observed midlevel-hump pattern. The Masking Condition × Standard Level interaction was not significant, *F*(2, 14) = 1.97, *p* = 0.324. Thus, contrary to expectation, facilitating selective attention to the target via auditory grouping was not of greater help in the “midlevel” condition (standard level 60 dB SPL), although descriptively the data indicate such an effect.

The detection thresholds in quiet and in the three conditions presenting forward maskers are displayed in **Figure 3**B. A repeated-measures ANOVA conducted on the data obtained under masking showed no significant effect of the number of maskers, *F*(2, 14) = .02, *p* = .983. This confirms our assumption that the 3 M condition does not produce stronger (or weaker) adaptation in the auditory periphery than the 1 M condition.

### Experiment 2: Masker Stream

**Figure 4:**
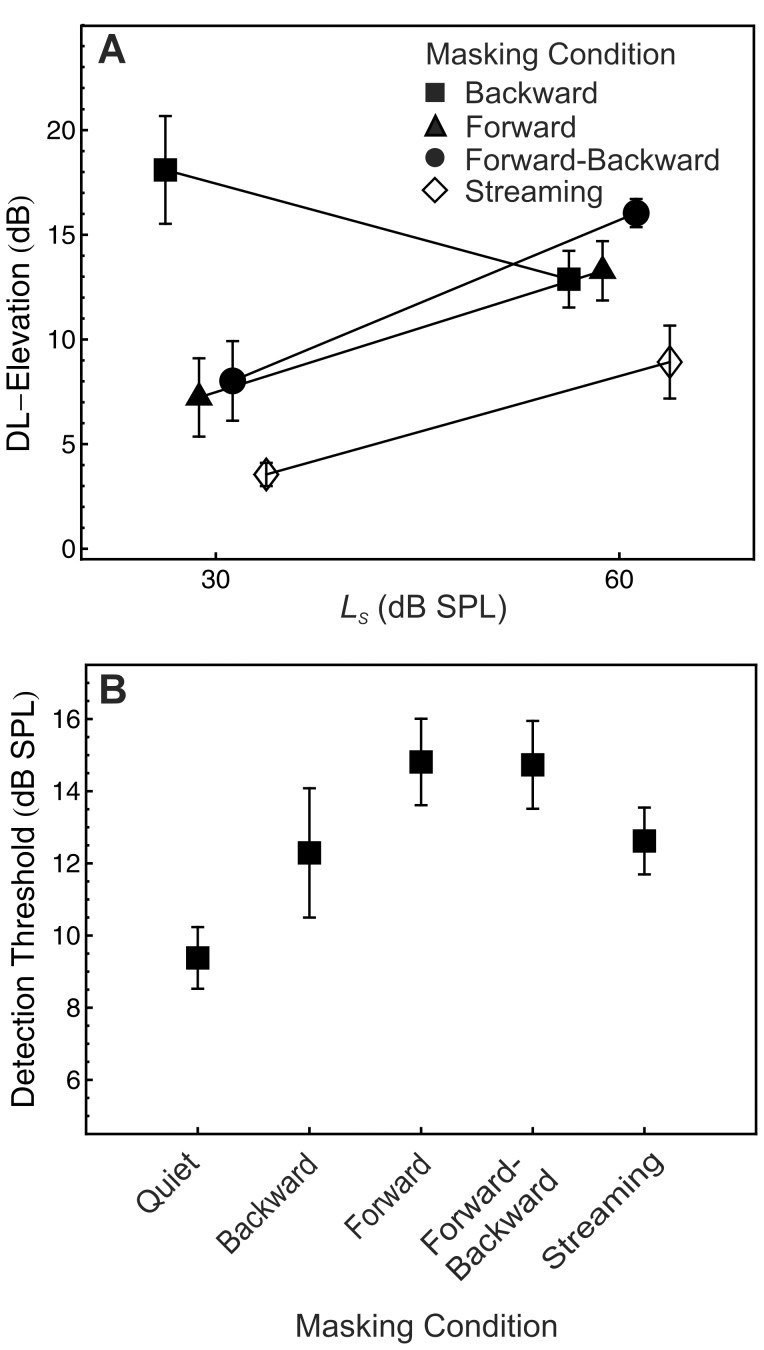
Experiment 2: intensity resolution and detection thresholds. Panel A: mean DL-elevation as a function of standard level (*L*_S_) and masking condition. Boxes: backward masker. Triangles: forward masker. Circles: forward and backward masker. Open diamonds: streaming condition. Panel B: Mean detection thresholds in quiet and in the four masking conditions. Error bars show ±1 SEM. *N* = 7.

The mean DL-elevations observed in Experiment 2 are displayed in **Figure 4**A, for the four masking conditions and the two standard levels. For all masking conditions except backward masking, the DL-elevation was higher at a standard level of 60 dB SPL than at a standard level of 30 dB SPL, thus demonstrating a midlevel hump [36]. A repeated-measures ANOVA with the within-subjects factors standard level and masking condition showed that this effect of standard level was significant, *F*(1, 6) = 8.91, *p* = .024, *η^2^* = .60. The mean DL-elevation was minimal in the streaming condition at both standard levels, which is compatible with our hypothesis that the DL-elevation in the streaming condition is reduced because maskers and targets are perceived as two separated objects. This observation was confirmed by a significant effect of masking condition, *F*(3, 18) = 24.00, *p*<.001, 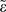 = .77, *η^2^* = .80. The Standard Level × Masking Condition interaction was also significant, *F*(3, 18) = 5.69, *p* = .007, 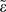 = .99, *η^2^* = .49, most likely owing to the unexpectedly high DL-elevation observed in the backward masking condition at the 30-dB SPL standard level. Note that several previous studies also reported extremely high DL elevations for some listeners if a low-level standard was combined with an intense masker (for a discussion see [10]). At the 60-dB SPL standard level, the effect of forward and backward masking was nearly identical, again compatible with previous studies [8]. Was the significant effect of masking condition reported above simply caused by the unexpectedly high DL elevation under backward masking at the lower standard level, rather than by the expected difference between the streaming condition and other conditions? To answer this question, we conducted an additional two-factorial ANOVA with the data from the backward masking condition excluded, which again showed a significant effect of masking condition, *F*(2, 12) = 21.50, *p* = .001, 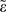 = .76, *η^2^* = .78. Compared to the forward masking condition, the streaming condition resulted in a release from masking of 4.02 dB (*SD* = 3.14 dB, *d_z_* = 1.28). The streaming condition caused an even stronger release from masking of 5.79 dB (*SD* = 1.79 dB, *d_z_* = 3.32) if compared to the forward-backward masking condition. As expected, in the forward-backward condition the DL-elevation was 1.77 dB (*SD* = 2.03 dB, *d_z_* = 0.87) higher than in the forward masking condition. Post-hoc pairwise comparisons indicated that the DL-elevation in the streaming condition differed significantly from the forward masking and the forward-backward condition, while the difference between the latter two conditions was not significant. The significant difference between the DL-elevation in the streaming and in the forward-backward masking condition shows that the smaller masking effect in the streaming condition cannot be attributed to the presence of two rather than one adjacent maskers. In the ANOVA with the backward masking condition excluded, the standard level × masking condition interaction was non-significant, *F*(2, 12) = 0.36, *p* = .703.

On average, detection thresholds were highest in the forward masking condition and in the forward-backward masking condition (see **Figure 4**B), followed by the thresholds under backward masking and in the streaming condition. A repeated-measures ANOVA showed a significant effect of masking condition, *F*(4, 24) = 10.40, *p* = .001, 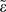 = .65, η^2^ = .63. Post-hoc pairwise comparisons between all pairs of masking condition including the in quiet condition were conducted. For the total of 10 pairs tested, only four tests were significant (α = .05, Hochberg procedure). In all masking conditions expect backward masking, the thresholds were significantly higher than in quiet. This pattern is compatible with current models of forward masking (e.g., [46]). Surprisingly, however, the detection threshold in the streaming condition was significantly lower than in the forward masking condition, probably indicating an effect of grouping even for the detection task.

In Experiment 2, we obtained information about the subjective perceptual organization of the stimuli at the end of the final session. In the streaming condition, each listener provided one rating of the perceived organization at the lower standard level and one rating at the higher standard level. Listeners indicated to have perceived the maskers and the targets as separate groups in 11 of the 14 cases (7 listeners × 2 standard levels). One listener reported to have perceived maskers and targets to be grouped together at the 30 dB SPL standard level, but as segregated at the 60 dB SPL standard level. One other listener made use of the opportunity to draw in the perceived organization of stimuli at both standard levels. Unfortunately, we were not able to classify the drawings as either indicating integration or segregation of maskers and targets. Taken together, the subjective ratings are compatible with our assumption that the streaming condition favored the processing of the maskers and the targets as two separate auditory objects, despite the fact that we used a rather short duration of the masker sequence (e.g., [56]). The results were not quite as clear cut for the remaining masking conditions. The tones presented in an observation interval were perceived as grouped together 19 times, and thus in only 45.2% of the 42 non-streaming cases (7 listeners × 3 masking conditions × 2 standard levels).

## Discussion

We measured intensity resolution for pure tones in the presence of non-simultaneous maskers. By comparing conditions either favoring the perception of masker and target as one unitary object, or as two separate objects, we tested the hypothesis that limitations of selective attention play an important role for intensity discrimination performance. Compatible with our hypothesis, compared to the usual forward masking setting we found a significant release from masking of 3 to 4 dB if the maskers could be grouped together on the basis of temporal proximity (Experiment 1), or could be perceived as a separate auditory stream (Experiment 2). These results are compatible with the assumed role of object-based attention for intensity resolution under non-simultaneous masking.

Two potential problems with this proposed interpretation of our results should be noted. First, although we presented conditions likely to favor the grouping of maskers and targets into separate objects, these manipulations may not have had the desired effect. Second, the question could be asked whether processes different from selective attention might account for the results, for example because the masking conditions may have differed in terms of peripheral adaptation.

Concerning the question of whether our manipulations of the masker-target sequence indeed caused different perceptual organizations, the reports of the subjects in Experiment 2 indicated that the streaming condition favored the perceptual of maskers and targets into separate objects, compared to the remaining conditions. In Experiment 1, unfortunately, we had failed to collect subjective ratings of the perceived organization. Thus, while it appears likely that in the 3 M conditions the three maskers were perceived as one object due to their temporal proximity [15], we have no definite empirical evidence for it. This aspect should be improved in future experiments, although it should be noted that many previous studies concerned with effects of grouping, for example in the context of comodulation masking release (e.g., [57]), informational masking [58], [59], or visual attention [20], did not explicitly measure the perceived grouping, either.

Could the observed release from masking in the 3 M condition (Experiment 1) and the streaming condition (Experiment 2) be attributed to other mechanisms than the facilitation of selective attention to the targets? Evidently, it is critical that experimental manipulations intended to either favor or discourage perceptual grouping of maskers and targets into separate objects should not cause effects in the auditory periphery that could confound the changes in intensity resolution expected due to object separation. For example, frequency differences are one of the strongest cues favoring auditory stream segregation [22], [49], [60]. However, increasing the frequency difference between masker and target will also cause the two tones to activate different locations on the basilar membrane (cf. [61]). As a consequence, peripheral adaptation effects, for example in auditory nerve neurons, would be reduced by the frequency separation between masker and standard (e.g., [62]). Thus, it is difficult to decide whether reduced peripheral adaptation, or a facilitation of selective attention due to grouping, or both are responsible for the reduction in the masker-induced DL-elevation observed if masker and standard differ in frequency [9]. For this reason, we presented conditions that could be expected to improve intensity resolution due to perceptual grouping, while not causing a potentially confounding reduction in adaptation at early processing stages. This assumption is corroborated by the detection thresholds measured in Experiment 1, which showed no significant differences between the three masking conditions. Therefore, it seems very unlikely that the observed *reduction* in the masker-induced DL-elevation in the 3 M condition compared to the 1 M condition could be attributed to a reduction in adaptation at early stages. Additionally, the three maskers presented in the 3 M condition did not render the standard undetectable, which might have led to different decision strategies in the intensity discrimination task [63]. In Experiment 2, the stimulus characteristics and the critical temporal parameters were also held constant across conditions. In the latter experiment we observed a beneficial effect of grouping not only in the discrimination task but surprisingly also in the detection task. In our opinion, this does not necessarily imply that the streaming condition led to superior processing at the earliest stages, however, because recent studies suggest that psychophysical detection performance cannot be explained solely on the basis of auditory nerve adaptation [64], [65]. Therefore, streaming might have an effect in higher processing stages involved in a detection task [66], [67].

In both experiments of the present study, the smallest masker-induced DL-elevation was observed in the condition presenting the highest numbers of maskers. Could the DL-elevation simply decrease with the number of maskers? The data from Experiment 2 argue against this explanation. The effects of masking first increased with number of maskers when going from the forward masking condition to the forward-backward condition, but then decreased again in the streaming condition presenting the highest number of maskers. Additionally, if the intensity resolution would increase with the number of tones per trial, then the worst performance should be observed in quiet, which is clearly not the case. From a physiological point of view, while neural response enhancement by previous tones has been reported (e.g., [68], [69], [70]), some studies found that a spectral difference between consecutive tones is required for neural response enhancement by previous sounds (e.g., [71]). Because in our experiments the frequencies of maskers and targets were either identical or very similar, an enhancement of the neural response to the targets by preceding maskers seems unlikely.

If one compares the temporal sequence of stimuli between the forward-backward masking condition and the streaming condition, it is difficult to imagine that in the forward-backward condition the missing “inducing” tones or the missing middle masker should have had an effect other than on the formation of a “masker stream”. These tones missing in the forward-backward condition were separated from the target by more than 350 ms in the streaming condition. For this temporal separation, the maskers’ effect on the neural representation of the target in the auditory nerve should be very weak [9], [72]. Even if the additional maskers in the streaming condition would have resulted in stronger adaptation in auditory nerve neurons, this should have caused an impairment in performance, rather than the observed *release* from masking.

Taken together, after considering other potential explanations for our results, it appears reasonable to take our findings as evidence of the modulation of selective attention to the target by the perceived grouping of maskers and targets. An explanation of the effect of non-simultaneous masking on intensity resolution based on selective attention (or rather, on the failure of selective attention) is directly compatible with reports that the *perceptual similarity* between the masker and the target is an important factor in predicting the masker-induced DL elevation (e.g., [10], [38], [73]). For instance, adding a 4.133-kHz “cue tone” to a 1-kHz forward masker was found to strongly reduce the size of the midlevel hump for a 1-kHz standard [73], in terms of our proposed concept presumably by promoting the grouping of target and masker into separate auditory objects (see below). This finding is difficult to explain in terms of some of the three models discussed above. For example, according to the model proposed by Zeng *et al.* [36], the cue tone should produce additional adaptation in the auditory nerve, although predominantly in neurons tuned to frequencies well above the target frequency. In any case, this would not explain the *improvement* in performance observed with the cue tone. Within the framework of selective attention, accounting for effects of the masker-target similarity is no problem, as for instance widely accepted explanations for the effects of the target-distractor similarity in visual search tasks demonstrate (e.g., [74]). Note that the midlevel hump and the mid-difference hump might also be considered as an effect of masker-target similarity on the loudness dimension [10]. Accounting for the effects of backward maskers is straightforward in terms of selective attention: the backward maskers represent “distractors”, just as forward maskers do. Finally, if we assume that the limited capability of assigning selective attention to the target is the cause of the masking effects, then within-trial variations in masker level should generally have an effect on the response. Compatible with this prediction, Oberfeld [27] reported that the to-be-ignored masker intensity had a systematic influence on the decision in a forward-masked intensity discrimination task. Taken together, the framework of selective attention has the potential to integrate previous finding on effects of non-simultaneous masking on intensity discrimination.

The results from Experiment 1 and 2 are compatible with recent data from our lab where the lateralization a binaural forward masker was varied via the inter-aural time difference (ITD) so that it was lateralized either on the same side of the head as the binaural target, or on the opposite side [75]. On average, the DL-elevation was 3.5 dB smaller for the contralateral than for the ipsilateral masker, similar to the average effect observed in the present study. Notably, because only the masker ITD was varied, the waveform delivered to each of the two ears (i.e., the monaural channels) was identical in the conditions with ipsilateral and contralateral masker. This ensured that the representation of masker and target in the auditory nerve did not differ between the two masker lateralizations. Therefore, the release from masking observed in [75] is compatible with the framework proposed here, according to which the object segregation promoted by the lateralization differences between masker and target facilitate object-based selective attention to the target.

It is interesting to note that in Experiment 2 we observed effects of grouping despite the fact that the stimuli may have favored stream segregation only to a rather limited extent. Sequential stream segregation is typically studied with pitch differences of several semitones (e.g., [22]). However, the presence of multiple cues to streaming (frequency, duration, intensity) may have enhanced streaming [76], [77]. Another finding of potential relevance is that in some studies the buildup of streaming was reported to be rather slow (e.g., [47], [48], [56]), so that the four maskers presented before the targets in the streaming condition might be viewed as the minimum sequence length resulting in stream segregation. Again, the availability of multiple cues could have accelerated the build-up. The ratings of the subjective organization of the stimuli obtained for Experiment 2 indicate that listeners indeed perceived the maskers as a unitary stream. Still, it would be interesting for future experiments to include conditions causing a higher amount of streaming. This would allow estimating the maximal release from masking that can be achieved by grouping maskers and targets into different auditory objects. Even more important, as one reviewer suggested, in longer tone sequences the perception as integrated or segregated is often bistable [78], and this effect could be exploited for comparing the intensity resolution on trials where the listener perceived the maskers and the targets as two segregated streams versus on trials where he or she perceived the sequence of tones as integrated. In this way, any potential confounds by for example different number of tones could be avoided.

As stated in the Introduction, the effect of object-based attention on intensity resolution indicated by the results is compatible with our proposal that the decision stage receives a precise representation of target intensity even under non-simultaneous masking, but makes only suboptimal use of it, due to limitations of selective attention [27]. For example, it might be the case that not only the two target intensities presented in the 2I task are compared with respect to intensity but that the decision is systematically influenced by masker intensity, as it was demonstrated in an earlier study from our lab [27]. We assume that if at the processing stages prior to the decision stage the maskers and the targets are grouped as separate objects, then the inclusion of masker information at the decision stage will be less likely (cf. [10]). Our proposal is compatible with comparisons between physiological and psychophysical effects of forward masking on intensity discrimination, which suggest that at least at early processing stages there is more information available than is reflected by the psychophysical data [65], [66], [79], [80]. It would be interesting to compare the intensity discrimination performance of simulated or real neurons at higher processing stages to our psychophysical data, for the experimental conditions we studied. Concerning the question of at which physiological structures the effects might be mediated a relatively strong constraint is imposed by the observation of strong effects of backward maskers on intensity resolution (see Experiment 2). At the neuronal level, this effect could be due to persistence of the neuronal response to the target for more than 100 ms. This ongoing activity could be terminated or reduced by the backward masker. Alternatively, if the maskers produced long-lasting inhibition of at least several hundred milliseconds, then the backward masker presented in the first observation interval might reduce the neural response to the target presented in the second interval. There is clear evidence for persistence over an interval of more than 100 ms and for inhibition for several seconds in the medial geniculate body and in primary auditory cortex [70], [81], [82], but most studies suggest that the two phenomena do not occur at earlier processing stages (for an in-depth discussion see [28]).

Attempts to identify neural correlates of auditory stream segregation have focused on the auditory cortex (e.g., [31], [83], [84], [85]). However, evidence for an involvement of earlier processing stages [86], [87], [88] and later processing stages [89] has also been reported. We believe that the effects of auditory object formation and object-based attention found in our experiments represent an important constraint for physiological models of intensity discrimination, especially because we observed effects of auditory grouping without varying the frequency difference between maskers and targets. Thus, the release from non-simultaneous masking found in the conditions favoring the perception of targets and maskers as separate objects cannot be explained simply by the tonotopic organization of the physiological structures responsible for the effects [90]. In fact, physiological correlates for streaming based on non-spectral cues were recently reported [91], [92].

Most previous studies on neural correlates of stream segregation looked for differential enhancements or reductions in the physiological responses to the two types of tones presented in the alternating type of sequences (e.g., ABAB…) frequently used for investigating auditory stream segregation [22]. For example, the frequency of the A tones was set to the best frequency for the recording site, and the frequency difference between the A and the B tones was varied. The decrease in neuronal activity with increases in the frequency separation between the A and B tones was compared to the percentage of trials on which human listeners reported the A and the B tones to form two separate streams [90]. The paradigm used in the present study represents a complement to this type of experiments because the changes in the intensity DLs found in our experiments are an objective rather than a subjective measure of auditory grouping [93]. Therefore, it would be possible to apply signal detection theory analyses to the neuronal responses [65] in order to identify neuronal correlates of psychophysical performance. Note that the stimuli and the task we used are relatively simple, so that they should be suitable for physiological and behavioral experiments in non-human subjects.

Electrophysiological data would also provide an opportunity to gain a deeper insight into the mechanisms responsible for the observed effects. Specifically, the mismatch-negativity (MMN; cf. [94]) has been used successfully for investigating auditory grouping (e.g., [47], [95], [96]). The effect of auditory grouping on the detection of changes in target intensity and on the involuntary shifting of attention towards the masker could also be assessed via MMN and other electrophysiological indices, for example in a distraction paradigm [97], [98].

Our results are in accord with evidence for effects of object-based attention on informational masking, which by definition also represents the effects of processes beyond the auditory periphery (cf. [99]). Sequential streaming has been reported to strongly reduce the amount of informational masking [58], [59]. For effects of the masker-target similarity, which were also demonstrated to play a strong role for informational masking (e.g., [100], [101]), Shinn-Cunningham [13] proposed that the similarity effects are mediated by auditory object formation, because “*(…) similarity can cause the target and masker to be perceived as part of the same, larger perceptual object (…)*”.

A recent study by Dyson and Ishfaq [102] provides support for object-based processing in auditory short-term memory. Retrieving two attributes originating from the same auditory object was faster than if the second attribute originated from a different object. These results indicate that objects play a similar role in auditory memory tasks as in visual memory tasks, in line with the analogy we assume between object-related attention processes in audition and in vision. Taken together, the concept of auditory selective attention linked to auditory object formation proposed here as a framework for understanding the effect of non-simultaneous masking on auditory intensity resolution is well grounded in previous research.
